# The Effects of Voluntary, Involuntary, and Forced Exercises on Brain-Derived Neurotrophic Factor and Motor Function Recovery: A Rat Brain Ischemia Model

**DOI:** 10.1371/journal.pone.0016643

**Published:** 2011-02-08

**Authors:** Zheng Ke, Shea Ping Yip, Le Li, Xiao-Xiang Zheng, Kai-Yu Tong

**Affiliations:** 1 Department of Health Technology and Informatics, The Hong Kong Polytechnic University, Hong Kong SAR, China; 2 College of Biomedical Engineering and Instrument Science, Zhejiang University, Hangzhou, China; University of North Dakota, United States of America

## Abstract

**Background:**

Stroke rehabilitation with different exercise paradigms has been investigated, but which one is more effective in facilitating motor recovery and up-regulating brain neurotrophic factor (BDNF) after brain ischemia would be interesting to clinicians and patients. Voluntary exercise, forced exercise, and involuntary muscle movement caused by functional electrical stimulation (FES) have been individually demonstrated effective as stroke rehabilitation intervention. The aim of this study was to investigate the effects of these three common interventions on brain BDNF changes and motor recovery levels using a rat ischemic stroke model.

**Methodology/Principal Findings:**

One hundred and seventeen Sprague-Dawley rats were randomly distributed into four groups: Control (Con), Voluntary exercise of wheel running (V-Ex), Forced exercise of treadmill running (F-Ex), and Involuntary exercise of FES (I-Ex) with implanted electrodes placed in two hind limb muscles on the affected side to mimic gait-like walking pattern during stimulation. Ischemic stroke was induced in all rats with the middle cerebral artery occlusion/reperfusion model and fifty-seven rats had motor deficits after stroke. Twenty-four hours after reperfusion, rats were arranged to their intervention programs. De Ryck's behavioral test was conducted daily during the 7-day intervention as an evaluation tool of motor recovery. Serum corticosterone concentration and BDNF levels in the hippocampus, striatum, and cortex were measured after the rats were sacrificed. V-Ex had significantly better motor recovery in the behavioral test. V-Ex also had significantly higher hippocampal BDNF concentration than F-Ex and Con. F-Ex had significantly higher serum corticosterone level than other groups.

**Conclusion/Significance:**

Voluntary exercise is the most effective intervention in upregulating the hippocampal BDNF level, and facilitating motor recovery. Rats that exercised voluntarily also showed less corticosterone stress response than other groups. The results also suggested that the forced exercise group was the least preferred intervention with high stress, low brain BDNF levels and less motor recovery.

## Introduction

There is enormous burden of stroke in many countries around the world, which brings about intense interests in studying effective rehabilitation training programs. Many studies have substantiated the beneficial effects of exercise on stroke recovery in animal models [1]–[4]. Among different exercise paradigms, voluntary wheel running, forced treadmill running, and involuntary muscle movement caused by electrical stimulation are the commonly adopted exercise models. Apart from their physical benefits, these exercises have been separately demonstrated to improve cognitive function and facilitate neural rehabilitation after brain damage [2]–[4]. It is important to know which rehabilitation intervention is more effective in facilitating motor recovery and up-regulating brain neurotrophic factor (BDNF) which is a leading factor in learning and memory, after brain ischemia.

Treadmill running has been applied widely as a rehabilitation scheme in both clinical studies and animal models. Stroke patients had lower energy expenditure and improved gross motor efficiency and would gain a better ambulation after treadmill exercise [5]. Many studies on animals also showed beneficial effects of treadmill exercise such as a smaller brain infarct volume or better neurological function either before [6] or after [3] stroke when compared with spontaneous recovery. Some studies, however, suggested that such beneficial effects only existed in low-intensity treadmill (15 m/min) running rats, and moderate-intensity treadmill (25 m/min) would elevate serum corticosterone [7]. Corticosterone is a typical sign of chronic stress, which usually causes reduced body weight and spleen atrophy [8], indicating a response of negative adaptation to stress. Moreover, corticosterone was shown to reduce BDNF availability in the rat hippocampus [9].

Wheel running is generally regarded as a type of voluntary exercise in animal models, and it does not activate systemic stress [10]. Although some research suggests that voluntary wheel running is not efficient in reducing brain infarct volume compared with forced treadmill running when performed before stroke [6], Marin at al. [2] concluded that there was no direct relationship between brain infarct volume and motor recovery. Moreover, other studies found that voluntary exercise showed superior effects in terms of plastic changes in the dentate gyrus [11], [12]. In accordance with this, Huang and his colleagues showed that up-regulation of BDNF lasted seven and two days in the wheel group and the treadmill group respectively [13].

Some involuntary exercise such as functional electrical stimulation (FES) by stimulating the paralyzed muscle by a specific stimulation pattern has also been involved in stroke rehabilitation program [14], [15]. It is found that by electrically stimulating affected limb, stroke patients had better motor recovery and walking ability when compared with the placebo group [14]. In animal studies, rats receiving such electrical stimulation during brain ischemia showed decreased infarct volume and better behavioral outcomes [4]. Besides, FES was also shown to up-regulate BDNF expression in stimulated muscles in rat models [16].

To date, effectiveness of voluntary, involuntary and forced exercises has not been fully compared under an animal brain ischemia model. This study is to investigate the functions of these three rehabilitation interventions in the regulation of stress response and brain BDNF expression. In parallel, we also compare their effects in facilitating motor recovery after brain ischemia in a rat model. The comparison results would help to provide more information for the clinical practice in the future.

## Methods

### Surgical Preparation

**Figure 1:**
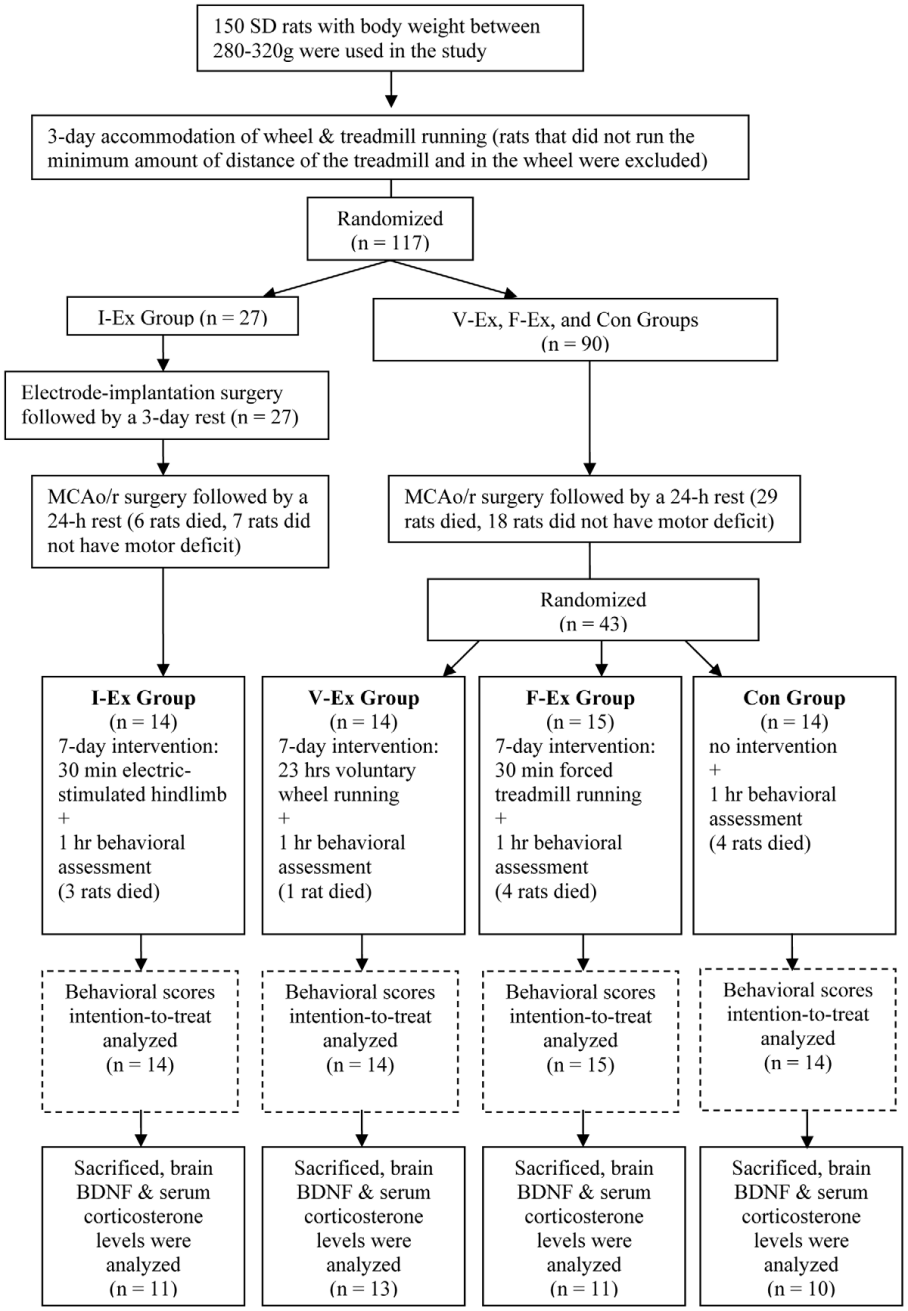
CONSORT flowchart of the experiment procedure. All rats were firstly accommodated to the wheel and treadmill exercises. I-Ex rats received an electrode-implantation surgery which generated the gait-like muscle contraction under controlled electrical stimulation. All rats were induced ischemic stroke with the MCAo/r surgery and received prescribed intervention 24 hours after the surgery. The intervention lasted for 7 days, and during which period behavioral test was conducted daily to assess the motor recovery level. After sacrifices, the serum corticosterone level and brain BDNF level were analyzed.

**Figure 2:**
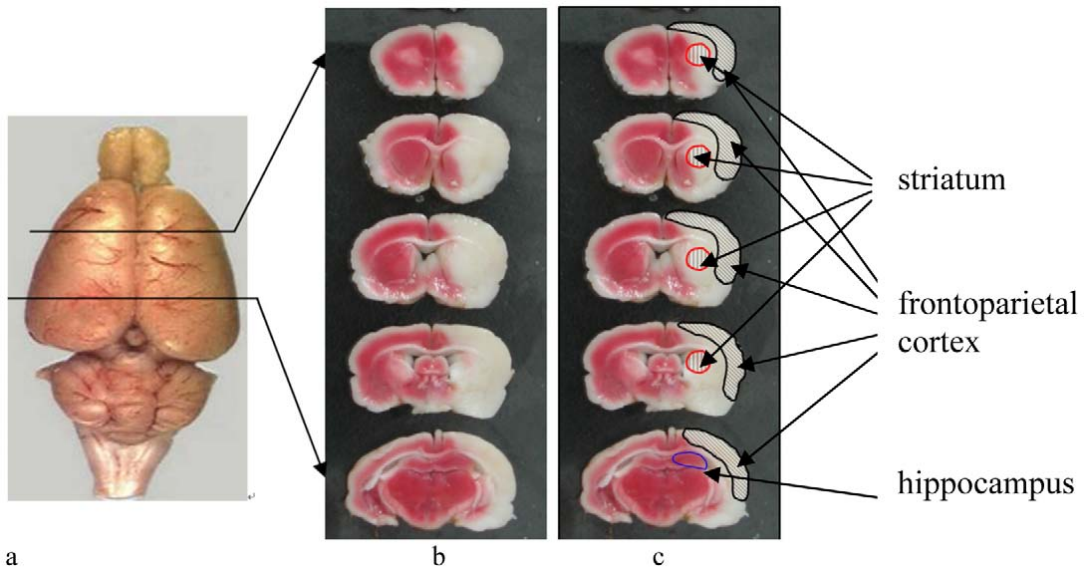
Brain infarction revealed by triphenyltetrazolium chloride (TTC) staining. (a) Rat brain (b) original TTC image of brain infarction after 90 minutes middle cerebral artery occlusion; and (c) image with labels. TTC reacts with dehydrogenases in viable cells and results in a “brick-red” color, and the white area indicates the infarction. It was clearly detected that ipsilateral motor cortex and striatum were affected with middle cerebral artery occlusion.

All procedures performed were approved by the Animal Ethics Review Committee of the University and conformed to international guidelines on the ethical use of animals. This study involved 150 young male Sprague-Dawley (SD) rats with body weight of 280–320g and provided by the Central Animal Facility of the University. All rats were allowed to access to food and drinks *ad libitum*. The detailed experiment procedures are shown in Figure 1. Briefly, all the rats were firstly trained to run on the treadmill and in the wheel as accommodation. The accommodation lasted for three days, and rats that failed to run the minimum amount of distance of the treadmill (600 m) and in the wheel (600 m) daily were excluded. Rats that met the minimum distance requirement after the accommodation period (n = 117) were randomly divided into two groups: 27 rats in the involuntary exercise group (I-Ex) with electrode implantation and received muscular electrical stimulation after stroke, and 90 rats in the other group without electrode implantation and received other rehabilitation interventions after stroke. Electrode-implantation surgery was carried out on the I-Ex after the rats were anaesthesized by 10% chloral hydrate (0.4 mg/kg for induction and 0.02 mg subsequently). Incisions were made on the skull to expose the bregma, and on the left hindlimb to expose the muscle pairs namely tibialis anterior (TA) and medial gastrocnemius (MG). Four teflon-coated stainless steel wires (AW633, Cooner Wire, USA) were passed subcutaneously from the skull to the incisions on the limb. One end of each wire was fixed by stripping insulation off and looping them around the bellies and tendons of TA and MG, respectively, and the other end was soldered to a 4-pin connecter and fixed on the skull with three screws, which were implanted on the surface of the skull, and fixed with dental cement [15]. After suture, thresholds of stimulation voltages that allowed TA and MG contraction were recorded and were 1.5–6 V and 2– 8 V respectively (S8800 Stimulator, Astro-Med, USA.) when the rat were still under anesthesia. After 3 days rest, I-Ex rats (n = 27) and other 90 rats were induced to suffer from ischemic stroke with intraluminal suture middle cerebral artery occlusion/reperfusion (MCAo/r) model under the same anesthesia method mentioned above [17]. The rat's right MCA was blocked by a 3-0 uncoated monofilament nylon suture with rounded tip for 90 minutes, and allowed reperfusion by withdrawing the suture. The damages produced in the brain included striatum and frontoparietal cortex (Figure 2), which were the areas in the brain that undertook BDNF evaluation after the rats were sacrificed in this study. Rats with successfully induced stroke (n = 57) were divided into four groups: I-Ex (n = 14), voluntary wheel exercise group (V-Ex, n = 14), forced treadmill exercise group (F-Ex, n = 15), and control group (Con, n = 14). Studies showed that 1-week exercise could significantly facilitate the motor recovery [2], modify the capacity of learning and memory, and BDNF regulation in the brain in rats [18], [19]. Based on these evidences, rats in this study were assigned to the three exercise groups and received their prescribed rehabilitation intervention for seven consecutive days (I1-I7), while Con rats were put in standard cage and allowed spontaneous recovery after stroke.

### Rehabilitation Interventions

**Figure 3:**
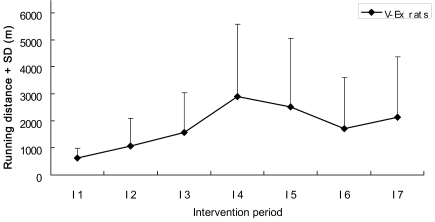
Exercise quantity of V-Ex rats. The quantity of exercise undertaken by the V-Ex rats is represented by their average daily running distance. The vertical bars in the graph represent the standard deviations (SD), while I1 to I7 represent the days of intervention after surgery. The running distance of V-Ex rats was similar as that of F-Ex rats (average 622.33 m vs 600 m) on I1, but gradually increased during the intervention period.

Twenty-four hours after the MCAo/r surgery, V-Ex rats were housed individually in a cage with a running wheel assembly and let free to run (manufactured by Kan Kee Sheet Metal Works, Hong Kong). The circumference of the wheel was 1 meter, and the wheel was connected to a switch that counted the number of revolutions [6]. The running distance of V-Ex rats was recorded and collated on a daily basis. F-Ex rats were forced to run on the motor-driven treadmill (KN-73, Natsume Ltd., China) at a speed of 20 m/min with a slope of 0° for a total of 30 minutes every day [20]. The 30-minute exercise was divided to three 10-minute sessions and with a 10-minute rest between successive running sessions. The rats would receive a nudge on the back when they did not catch up with the treadmill [21]. The running duration on the treadmill was based on a pilot experiment on rat wheel running and the experiment setup was intended to match the running distance between V-Ex and F-Ex. In this study, the distances were similar for V-Ex and F-Ex (average 622.33 m and 600 m, respectively) on the first day of intervention (I1), and the V-Ex rats increased running distance during their intervention period (Figure 3). I-Ex received a total of 30 minutes FES every day. Involuntary exercise of FES (I-Ex) with implanted electrodes in two hind limb muscles on the affected side was to mimic gait-like walking pattern during stimulation. The stimulation pulse was a biphasic rectangular pulse with a frequency 100 Hz, pulse width 300 µs and stimulation intensity was based on the muscle threshold for contraction during anesthesia [15]. The 30 minutes stimulation was divided to three 10-minute sessions and with a 10-minute rest between each session. The training and the rest periods in the stimulation protocol were planned to match with those of the F-Ex intervention protocol. The stimulation pattern (50 ms TA stimulation, 150 ms MG stimulation, and 300 ms rest) imitated the normal gait-like pattern of rat running on the treadmill at the speed of 20 m/min. The Con rats received no intervention and stayed in the standard cage. All rats were allowed to access to food and drinks *ad libitum* when they were not under training.

### Motor Function Recovery Tests

In this study, the De Ryck's behavioral test was applied to evaluate the motor function recovery after stroke. The behavioral test was conducted daily during the 7-day intervention period (I1-I7) and repeated for three times after everyday intervention and the average score was recorded. The behavioral test was based on the protocol of De Ryck et al., which is a 16-point scale including 8 tests with each scored from 0 to 2∶0 (maximum deficit), 1 (incomplete and/or delayed (>2 seconds) placing compared with the unaffected limb), and 2 (no deficit) [22]. Six out of 8 tests specifically evaluated forepaw's functions, including postural reflex, visual placing in the forward and sideways directions, tactile placing of the dorsal and lateral paw surfaces, and proprioceptive placing, while the other 2 examined the hindlimb's tactile placing of the lateral paw surfaces, and proprioceptive placing [4].

### Serum Corticosterone and Brain BDNF Evaluation

Serum corticosterone level and brain BDNF concentrations were determined with commercial enzyme-linked immunosorbent assay (ELISA) kits because ELISA is a sensitive and common tool for detecting these chemicals in rat tissues [6]. All rats were sacrificed by decapitation under anesthesia within 2 hours of their last intervention and assessment. Trunk blood was collected immediately after decapitation and centrifuged to obtain the serum for corticosterone level determination by ELISA kit (Assay Design, USA). The brain infarction volumes were measured by triphenyltetrazolium chloride (TTC) staining method in our pilot study, and it was shown that cortex and striatum were affected after MCAo (Figure 2). After sacrifice, the rat's brain was obtained by removing the skull, and the cerebral cortex was carefully removed to extract the striatum and hippocampus. The striatum, the motor cortex of the affected area, and the hippocampus were obtained to assess the levels of BDNF by E_max_® ELISA kit (Promega, Wisconsin, USA). All procedures were carried out according to the manufacturer's instructions.

### Statistical Analysis

Results were expressed as means ± SD. SPSS (version 15.0) was used for statistical analyses in this study. One-way analysis of variance (ANOVA) with Bonferroni post-hoc test was used to compare the serum corticosterone level, and the BDNF levels in the hippocampus, striatum, and cortex among four groups. In the comparison based on the behavioral test, multivariate analysis of covariance (MANCOVA) incorporating all outcome measures recorded from I1-I7 was used to reduce the probability of type I error owing to multiple comparisons [23]. This is a technique for assessing group differences across multiple metric-dependent variables simultaneously, based on a set of categorical variables as independent variables. The within-subject factor was set as time and the between-subject factor was set as group. The I1 measurement of each respective outcome was entered as covariate. If the MANCOVA revealed a significant effect, post-hoc analysis using Bonferroni correction was used to indicate significant differences between particular groups. The level of statistical significance was set at 0.05.

## Results

A total of 57 ischemic stroke rats with motor deficits were assigned into four groups, and 45 of these rats finished the 7-day intervention. Twelve rats that died during the intervention period were not included in the BDNF and corticosterone analysis, and the intention-to-treat method was used to analyze their behavioral scores, which means that the scores of their unfinished days were the same as that of the last score that we recorded on the day before it died. Table 1 shows the overall results of the behavioral test, the serum corticosterone concentration, and the brain BDNF levels in the hippocampus, cortex, and striatum. Significant differences were revealed in all the tests.

**Table 1:** Comparison of Motor Function Recovery, Brain BDNF levels, and Stress Response Outcome.

Variable	Group	Pre-intervention (I1)	Post-intervention (I7)	*P*	Post hoc(*P*)
Behavioral Score				<0.0001*	V-Ex & I-Ex(0.014*)
	V-Ex	4.70±1.16	11.90±1.20		V-Ex & F-Ex(<0.0001*)
	I-Ex	5.18±1.33	10.54±1.97		V-Ex & Con(<0.0001*)
	F-Ex	5.63±1.03	9.73±1.56		I-Ex & F-Ex(0.808)
	Con	4.00±0.82	7.90±1.29		I-Ex & Con(0.351)
					F-Ex & Con(1.000)
Hippocampal BDNF Level(ng/g)		-		<0.0001*	V-Ex & I-Ex(0.652)
	V-Ex		96.48±29.36		V-Ex & F-Ex(<0.0001*)
	I-Ex		83.05±13.90		V-Ex & Con(<0.0001*)
	F-Ex		18.29±5.92		I-Ex & F-Ex(<0.0001*)
	Con		51.78±6.08		I-Ex & Con(0.008*)
					Con & F-Ex(0.015*)
Striatal BDNF Level(ng/g)		-		0.005*	V-Ex & I-Ex(0.461)
	V-Ex		25.10±10.39		V-Ex & F-Ex(0.478)
	I-Ex		35.32±7.93		V-Ex & Con(0.857)
	F-Ex		14.96±5.09		I-Ex & F-Ex(0.007*)
	Con		16.45±9.78		I-Ex & Con(0.019*)
					Con & F-Ex(1.000)
Cortical BENF Level(ng/g)		-		0.035*	V-Ex & I-Ex(1.000)
	V-Ex		17.40±8.93		V-Ex & F-Ex(0.584)
	I-Ex		21.00±8.02		V-Ex & Con(1.000)
	F-Ex		12.15±4.88		I-Ex & F-Ex(0.053)
	Con		13.23±7.43		I-Ex & Con(0.141)
					Con & F-Ex(1.000)
Serum Corticosterone Concentration(nmol/l)		-		<0.0001*	F-Ex & V-Ex(<0.0001*)
	V-Ex		223.71±73.79		F-Ex & I-Ex(<0.0001*)
	I-Ex		394.72±83.70		F-Ex & Con(<0.0001*)
	F-Ex		656.51±156.57		I-Ex & Con(0.001*)
	Con		211.13±47.17		I-Ex & V-Ex(0.001*)
					Con & F-Ex(1.000)

Values: mean±SD; *P* value: significance level of MANCOVA multiple comparisons with covariates for behavioral score; significance level of one-way ANOVA for BDNF levels and corticosterone concentration.

*Indicates significant differences were revealed; post hoc was performed to specify the effect of group difference.

### Motor Function Recovery Outcome

**Figure 4:**
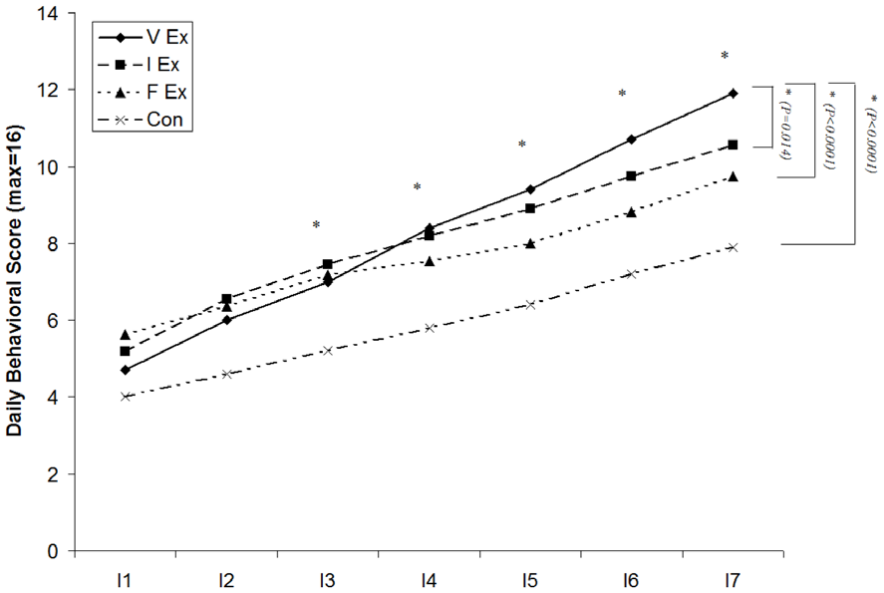
Daily behavioral score changes of four rat groups during intervention period. The motor recovery is evaluated with daily behavioral score of the V-Ex (♦), I-Ex (▪), F-Ex (Δ), and Con (x) during the 7-day intervention. *:a significant difference is revealed with MANCOVA from I3, and at the end of the training period V-Ex has significant higher behavioral score than I-Ex, F-Ex and Con in the post-hoc, indicating that voluntary exercise is more effective in facilitating motor recovery than involuntary exercise, forced exercise, and spontaneous recovery. Significant difference is not found between other groups after the 7-day intervention.

The motor function recovery outcome was presented as the behavioral score change and is shown in Figure 4. Significant differences among the four groups were found from day I3 and lasted until the end of the intervention period in the behavioral score (MANCOVA *P<0.0001*). At the end of the intervention, V-Ex had significant higher behavioral test score than I-Ex (*P = 0.014*), F-Ex (*P<0.0001*), and Con (*P<0.0001)*.

### Brain BDNF Levels and Serum Corticosterone Concentration

**Figure 5:**
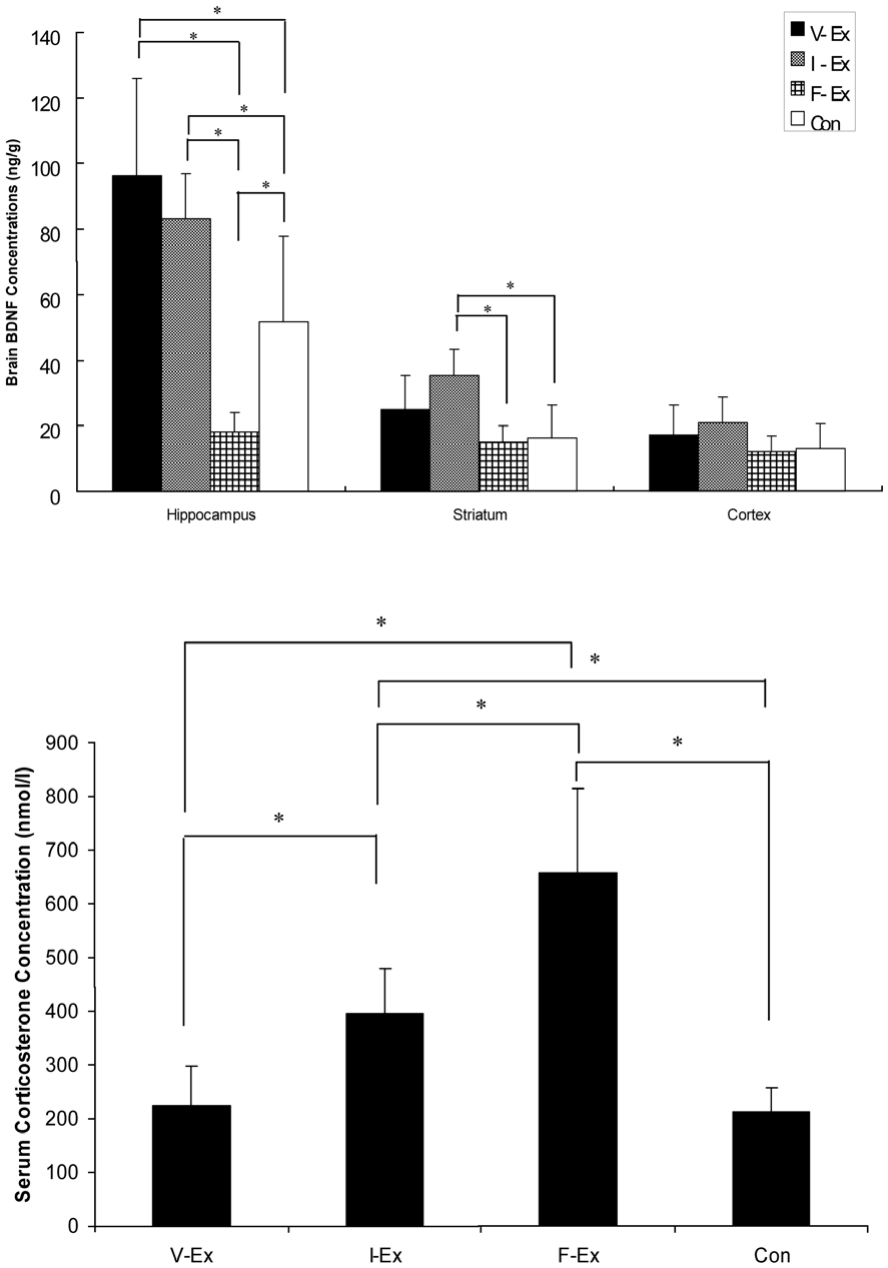
Comparisons of neurological status of four rat groups after sacrifice. The neurological status is evaluated with brain BDNF concentrations (A), and stress response was detected with serum corticosterone level (B). Values shown are mean ± SD *: a significant difference was revealed with one-way ANOVA. The V-Ex has a significant higher hippocampal BDNF level than other groups and a relative low corticosterone level as well. In contrast to V-Ex, F-Ex has the lowest hippocampal BDNF level and highest corticosterone level. This phenomenon might reveal a relationship between hippocampal BDNF level and serum corticosterone level.

The brain BDNF levels are shown in Figure 5A. Both V-Ex and I-Ex had higher hippocampal BDNF concentration than F-Ex and Con. Besides, I-Ex had significantly higher striatal and cortical BDNF concentrations than F-Ex and Con. The serum corticosterone concentration is shown in Figure 5B. F-Ex exceeded other three groups in the serum corticosterone concentration, and its average corticosterone concentration was around 3 times of those of V-Ex and Con (656.51 nmol/l vs 223.71 nmol/l & 211.13 nmol/l respectively). I-Ex also had significantly higher serum corticosterone concentration than V-Ex and Con.

## Discussion

This study is intended to compare the effectiveness between voluntary, involuntary, and forced exercises under similar training intensity with the evaluation of the motor recovery, and their functions of regulating corticosterone and brain BDNF levels after brain ischemia in a rat model. Our results showed that all three types of exercise applied in this study had better motor behavioral score than the control group. The voluntary exercise induced higher hippocampal BDNF level, better improvement in motor recovery when compared with involuntary exercise, forced exercise, and control groups. Moreover, our results also demonstrated that forced exercise could induce stress, which probably down-regulates BDNF levels in the brain.

BDNF is a powerful differentiation factor distributed widely in the central nervous system (CNS), and its concentration is particularly high in the hippocampus [24]. Our results also showed that BDNF concentration was higher in the hippocampus than in the cortex and striatum for all four groups. BDNF has been crucially implicated in neuroplasticity and is present throughout our life to enable essential functions such as learning and memory [25]. Recent studies suggested that exercise-induced enhancement in learning and memory is dependent on an increased BDNF level in hippocampal BDNF level, and the behavioral recovery was also correlated with a cell proliferation in the rat hippocampus [26], [27]. Moreover, BDNF had also been proved to consolidate short-term memories into long-term memories [28]. In adult rodents, BDNF also showed characteristics of neuroprotection which promoted survival of hippocampal, striatal, and septal neurons in culture and *in vivo* by protecting the brain against such insults as focal brain ischemia [29]. Evidence from other studies has revealed the effectiveness of spontaneous wheel running in the up-regulation of BDNF in the rat hippocampus [13]. Our results also showed that the hippocampal BDNF level in V-Ex rats was significantly higher than those of the Con and F-Ex. As first reported by Vanderwolf in 1969 [30], voluntary exercise activates a persistent firing pattern (known as theta-rhythm) in the rat hippocampus, and this firing pattern is dependent on cholinergic and GABAergic neurons. Such theta bursts lead to the secretion of BDNF in the hippocampus [31]. These evidences might be related to our results that V-Ex had the highest hippocampal BDNF level, and the highest last-day scores of both behavioral and beam walking tests. Our results also indicated that the motor recovery seems to be more related to the hippocampal BDNF level than the striatal and cortical BDNF levels, as V-Ex had high hippocampal but low striatal and cortical BDNF levels.

In this study, the results showed that I-Ex rats had the highest striatal and cortical BDNF levels. This might be attributed to the fact that electrical stimulation of the affected muscle groups brings about a series of physiological effects, including an increase in metabolism and cerebral blood flow (CBF) [16]. Electrical stimulation of peripheral nerves induces contraction of innervated skeletal muscles via motor nerve fibers, which consequently activates the CNS via sensory nerve fibers. With the stimulation of CNS, an increase of CBF is expected, and such linkage between neuron activities and CBF is also called activation-flow coupling. Burnett et al. demonstrated that, with the electrical stimulation of the rat's forepaw, the activation-flow coupling response was preserved over a broad range of baseline flow values during the MCAo/r [4]. As a result, a reasonable explanation is that electrical stimulation increases CBF, which consequently activates neurons and up-regulates chemicals such as trophic factors in these neurons.

The F-Ex rats had the lowest BDNF levels in the hippocampus, striatum and cortex and also the highest serum corticosterone level. This was probably due to the effects of stress on the F-Ex rats when they were forced to run. Forced exercise elevates the level of serum corticosterone [13], which decreases the availability of BDNF in the brain [8]. Compared with a low-dose subcutaneous injection of corticosterone in adrenalectomized rats, a high-dose injection gives rise to a substantial increase in the occupation of glucocorticoid receptors. It has been suggested that downregulation of BDNF expression in the hippocampus by corticosterone is mainly mediated by glucocorticoid receptors. Other studies have suggested that stress during treadmill training counteracted the beneficial effects of exercise on the up-regulation of BDNF. Cechetti and his coworkers found that a two-week moderate (20 min/day) treadmill training protocol did not alter the BDNF level in the rat hippocampus [32]. Considering the significantly higher serum corticosterone level and extreme low brain BDNF levels in the F-Ex rats, we speculate that corticosterone released during the forced exercise had downregulated the brain BDNF expression.

The results of the present study demonstrated that voluntary exercise was the most effective intervention in facilitating motor recovery, followed by involuntary exercise through functional electrical stimulation. The results also showed that voluntary exercise could greatly up-regulate the hippocampal BDNF level, and suppress the stress effects. Forced treadmill exercise was shown to elevate serum corticosterone concentration, which probably consequently reduced BDNF concentration in the rat brain, and it might diminish the beneficial effect of training on persons after stroke. Future studies may explore the effects of these exercise interventions in the infarct volume and neurogenesis.
